# Festival Dinner of Poplar Hospital

**Published:** 1924-01

**Authors:** 


### FESTIVAL DINNER OF POPLAR HOSPITAL.

"IT is the proud boast of the Poplar Hospital for Accidents that it has never been in debt," Lord Ritchie, the joint-chairman of the Hospital with Sir Joseph Broodbank, told the large gathering at the sixty-eighth annual festival dinner, over which he presided at the Holborn Restaurant, on December 19. Lord Ritchie had some interesting things to say about the work of the Hospital during the past year. Several alterations and improvements have taken place. A new entrance for patients has been made by which all cases can be taken direct into a courtyard instead of having to be carried from the ambulances across the public pavement in view of morbid-minded sightseers. An operating theatre for out-patients enables the staff to deal with any urgent cases immediately instead of sending them on to the London Hospital; and rooms for X-ray, massage, and electrical treatment will add greatly to the efficacy of the Hospital. Owing to the Rent Restrictions Act, Lord Ritchie said, it had not been possible to build the urgently needed hostel for the nurses. Because of the alterations in the west wing they had been obliged to put the nurses in an iron and wooden shelter. This was only a temporary measure, and seeing the arduous duties of the nursing staff, the hostel must be provided, and he was sure the hospital would not appeal in vain for the cost.

### A Chimney Sweep's Gratitude.

The Poplar Hospital serves the region in the East End known as "Dockland," and it is interesting to give Sir Joseph Broodbank's testimony at the dinner to the appreciation of its activities by the workers themselves. Last year, Sir Joseph said, working-men in that district had contributed £3,000 to the hospital by a self-imposed levy. He told this story. A chimney-sweep, through the skill of the hospital doctors and the care of its nurses, regained the use of a badly damaged arm. When he was well again he was engaged to sweep the chimneys. After nine months, as he asked for no payment, he was told to send in his bill, which ran: "To sweeping chimneys at Poplar Hospital, January to September, 1923. Settled with thanks for benefits received." Close upon £10,000 had been received in answer to a special appeal for the new work and the Nurses' Hostel. During the dinner Lord Ritchie announced that the £10,000 had been reached. Lord Ritchie, Sir Joseph Broodbank, Lord Southborough, who proposed "The Medical and Nursing Staff," and Mr. Russell Howard, the senior surgeon, who replied to Lord Southborough's toast, each expressed their admiration of the work done by the matron of the hospital, Miss Bland. Since she went to the hospital the beds have increased from sixty-three to a hundred and four.

